# Assessing rural populations’ barriers to mental healthcare and perceptions towards prescription digital therapeutics: a cross-sectional survey

**DOI:** 10.3389/fdgth.2025.1655446

**Published:** 2025-09-04

**Authors:** Grace Danon, Cody E. Dunn, Michael Robins, Arundati Nagendra, Chuck Strand, Lisa Palko, Adam Colborn, Jackie Menjivar, Junko Saber

**Affiliations:** ^1^ Innopiphany LLC., Irvine, CA, United States; ^2^ Schizophrenia and Psychosis Action Alliance, Alexandria, VA, United States; ^3^ Rural Minds, Mayville, NY, United States; ^4^ Society for Digital Mental Health, Irvine, CA, United States; ^5^ Academy of Managed Care Pharmacy, Alexandria, VA, United States; ^6^ Mental Health America, Alexandria, VA, United States

**Keywords:** prescription digital therapeutics, mental health, rural health, barriers to care, digital health, mental health equity

## Abstract

**Introduction:**

Prescription Digital Therapeutics (PDTs) hold unique potential to improve mental health in underserved rural areas. However, potential users' perceptions towards PDTs and community-specific differences in barriers to care are not well-understood.

**Methods:**

We conducted an online survey of 351 U.S. adults with ≥1 mental health condition and care-seeking behaviors. Descriptive statistics and non-parametric tests were used to evaluate rural and non-rural differences in demographics, social determinants of health, current barriers to mental health treatment, and the perceived value of PDTs. Key limitations of this approach include self-reported rurality and digital access bias associated with online survey distribution.

**Results:**

Barriers to mental healthcare impacted 60% of all rural respondents, and rurality was associated with unique challenges like lower incomes, lower education levels, substantial Medicaid enrollment, and further distances from care. Rural respondents were also more likely to be completely unfamiliar with digital apps for mental health treatment. 89% of all respondents thought PDTs could address at least one barrier to care and about 97% of respondents were likely to use a PDT recommended by their provider.

**Discussion:**

Existing gaps in care and positive perceptions towards PDTs demonstrate unique promise for these modalities to address unmet mental health needs. However, lower PDT familiarity among rural respondents suggests a need for provider intervention and policy reforms.

## 1 Introduction

In the U.S., 23% of adults—nearly 60 million Americans—reported experiencing a mental illness in the past year, yet over half (54.7%) did not receive treatment (1, 2). Complex environmental and social factors contribute to this shortfall, and certain communities experience inequitable impacts (3). This includes the 20% of Americans living in rural areas, who often face gaping disparities in mental healthcare access stemming from a milieu of infrastructural and sociocultural factors (4–6). Sixty-five percent and 47% of rural counties lack a resident psychiatrist and psychologist, respectively, compared to 27% and 19% of metropolitan counties (7). Securing transportation to this physically distant care may be especially burdensome for those who lack public transit or depend on others for their transportation (8). Even telemedicine remains unfeasible for some rural areas, where broadband may be limited or entirely unavailable (9–12).

Financial factors like lower socioeconomic status, higher unemployment, higher utilization of public health insurance, or lack of insurance altogether further impede rural mental healthcare access (10, 13, 14). These elements translate to reduced access to health information which deters appropriate and effective mental healthcare (15). In parallel, rural sociocultural influences—such as expectations of self-reliance, stigma associated with mental health, and less privacy in smaller communities—function as individual-level deterrents to seeking care (6, 16–18). Low treatment rates and poor care management as a result of these cultural influences and resource constraints point to the inadequacy of current modalities and a need for innovative alternatives that diversify care for rural communities (6, 19).

One proposed solution is Prescription Digital Therapeutics (PDTs): remote, software-based treatments that deliver clinically validated therapies on devices like smartphones or tablets (20). Unlike direct-to-consumer digital health apps, PDTs must be 1. tested in clinical trials, 2. reviewed by a regulatory body [e.g.*,* the United States Food and Drug Administration (FDA)] for efficacy and safety, and 3. prescribed by a healthcare provider (21). Currently, the FDA has cleared over 20 PDTs targeting a variety of conditions like irritable bowel syndrome, chronic lower back pain, and diabetes (22, 23).

Mental health is an especially promising application for PDTs given the demonstrated efficacy in this area, clear regulatory pathways, and opportunity to alleviate provider-patient imbalances (21, 24). Used independently or in combination with pharmacotherapy or cognitive-behavioral therapy, mental health PDTs are designed to provide evidence-based treatment in a convenient, user-friendly, and personalized format. Their scalability and ability to reduce in-person provider visits could offset treatment costs, while continuous monitoring and outcomes tracking may streamline care (22, 25). In this way, PDTs could be especially useful for rural populations who might benefit from fewer in-person interactions and additional support between appointments (3, 26, 27). With rigorous data and privacy standards in place, the option to be treated anonymously in one's own home may mitigate rural undertreatment stemming from mental health stigma (26).

However, similar to telemedicine, PDTs that depend on smartphone use or internet connectivity may be less feasible for users in “digital deserts,” where broadband is limited or unavailable (28–30). In conjunction, lower digital literacy might hamper interest among historically disadvantaged groups or compromise effective engagement once prescribed (28). Privacy and data security concerns also hinder willingness to use PDTs, particularly among populations with intergenerational distrust of the medical system (29). Ensuring that digitization mitigates, rather than exacerbates, health disparities requires thorough consideration of the factors shaping access to care. Quantitative and qualitative studies have evaluated such barriers and facilitators to digital mental health use; however, these generally probed on a wide scope of interventions (e.g., general “health apps” or telemedicine) rather than FDA-cleared and physician-prescribed therapeutics (31, 32). Both features could serve as impactful drivers of value, necessitating evaluation both separately and within the context of broader digital health benefits (33, 34).

In addition, few studies have considered the impact of rurality on the perceived utility of PDTs, despite its clear ties to both mental health treatment and digital access (35). One exception is a study by Jongebloed et al., which identified strong rural engagement with digital health products in Australia, however, findings were limited by the sample's high baseline digital literacy (36). A second, more applicable example involved a small qualitative survey of U.S. veterans (*n* = 66), which observed an overall lack of awareness about “mental health smartphone apps” and lower receptivity among rural veterans (37).

In developing a quantitative approach, ex-U.S. studies comprised the most informative research for this purpose, likely owing to the enhanced integration of PDTs into mainstream care via centralized market access and reimbursement frameworks (38). A study in two German academic centers surveyed urology patients to assess age-based differences in the use of digital therapeutics and valued product qualities (39). Another German study evaluated use, adherence, acceptance, and perceived PDT efficacy among a small sample of rheumatology patients (40). While these were not specific to mental health or rurality, the statistical analyses performed in these studies serves as an illustrative precedent.

To optimize capture of the lived experience and prioritize the interests of those directly impacted by this research, a diverse group of mental health advocates and digital health experts provided input on the study's design and interpretation. Drawing from these advising co-authors' recommendations and the existing literature, we developed the first quantitative, U.S.-based study that 1. focused on mental health and existing barriers to care, 2. probed perceptions of PDTs specifically, rather than digital health more broadly, and 3. meaningfully explored the impact of community setting on PDT receptivity (31, 32). The objective of this research was to apply an advocacy-led, people-centered approach (i.e., focused on the priorities and needs of those affected by the research) to evaluate differences in the treatment experiences of rural and non-rural (i.e., urban and suburban) people living with a mental health condition and the perceived utility of PDTs across settings (41). Understanding these community-level differences will likely be critical for integrating PDTs into treatment decision-making.

## 2 Materials and methods

### 2.1 Sample and study design

An online survey was administered to a sample of 351 rural (*n* = 145) and non-rural (*n* = 206) U.S. adults residing in any state/region in December 2024. Inclusion criteria and survey questions were developed based on a targeted literature review and input from the advising co-authors.

Due to the dearth of relevant existing literature, the survey was powered *a priori* to compare rural and non-rural perceptions of PDTs using rural vs. urban perceptions of neurology telehealth visits as a proxy (*α* = 0.05, *β* = 0.2, Mann–Whitney *U* test) (42–44). Due to de-identification concerns associated with using zip codes, participants were asked to self-report their community type as rural, suburban, or urban based on simple definitions: “a small town or the countryside,” “a residential area near a city,” or “a medium or large city,” respectively. Per the recommendation of a rural mental health advocacy group, participants identifying as urban or suburban were combined into a single “non-rural” category based on the binary precedent of defining rural as “non-metro” i.e., not urban or suburban (45–47). The survey assessed rural and non-rural differences for the following: social determinants of health (defined as nonmedical factors that shape health outcomes) (48); familiarity with digital health technologies; current methods of receiving mental healthcare; challenges to receiving care; and interest in and perceived value of PDTs, including their potential to address barriers to care.

### 2.2 Data collection

We aimed to recruit a diverse sample of care-seeking individuals living with at least one mental health condition, while still including those who had fallen out of care. A 10-question, self-reported eligibility questionnaire (approximately 4 min) required participants to meet the following inclusion criteria: 1. 18 years of age or older, 2. living with at least one, but no more than three, of the following mental health conditions: depression; anxiety; attention deficit disorder (ADHD); bipolar disorder; schizophrenia, schizoaffective, or another primary psychosis disorder (first episode psychosis); post-traumatic stress disorder (PTSD); or substance use disorder, and 3. demonstrating care-seeking behaviors to ensure a baseline interest in treatment. People living with only conduct disorder (which occurs predominately in children and adolescents), eating disorder (more prevalent among adolescents), or “other” were not eligible for the survey, but could be included if 1–2 of the eligible conditions were reported (49, 50).

Recruitment minimums and maximums were also set to ensure diversity within the sample and alignment with the target population. The minimum number of rural participants (*n* = 135) was based on a power analysis, as previously mentioned. Other minimums were informed by a targeted literature search and anticipated patient availability. These included: ≥35 patients with schizophrenia, schizoaffective, or another primary psychosis disorder (first episode psychosis), ≤50 patients with only ADHD, ≤225 White-Only patients, ≥50 patients that are 20–30 min from the closest in-person medical care, ≥50 patients that are more than 30 min from the closest in-person medical care (51–53). ADHD onset, diagnosis, and treatment typically occur in childhood. However, a portion of ADHD diagnoses persist into adulthood, and ADHD is an especially viable therapeutic area for PDTs (20, 54–56). As a result, a maximum was imposed for individuals living only with ADHD rather than excluding this group altogether. Additionally, given the pervasive undertreatment of schizophrenia, schizoaffective, and other primary psychosis disorders, a minimum was set for these conditions to ensure adequate representation despite a relatively low prevalence and known recruitment difficulties (57). Finally, the criterion for participants living more than 30 min from their closest in-person medical care was loosened to ≥50 patients living at least 20 min from their closest in-person medical care due to challenges recruiting sufficient respondents (final count: *n* = 36 living 20–30 min from care; *n* = 14 living >30 min from care).

Eligible participants completed a 16-question survey (approximately 6–8 min) including multiple choice, forced ranking, and rating scales based on a modified Likert scale, binary selection, and optional free response. All eligibility and survey questions are provided in the Supplementary Materials.

A third-party vendor programmed and hosted the survey. Two patient networks were used to secure sufficient sample sizes in a timely manner. The survey vendor managed responses and ensured there was no overlap between the networks. The usability and technical functionality of the survey were tested prior to fielding. The survey was voluntary and available for one-time completion through an online platform among registered individuals within the networks, which prevented duplicate entries. Participants were provided with the survey's length and description; the investigator name; the purpose, risks, and benefits of the study; and data security measures. Participants who agreed to the Statement of Consent moved on to the eligibility questionnaire. Questions were not randomized, but the order of responses was randomized for select multiple choice and forced ranking questions. Participants had the option to change responses to previous questions at any point prior to survey submission. Upon survey completion, participants either received $10 or were compensated with rewards network incentives.

This survey was determined exempt by the WCG Institutional Review Board, and all third parties were ISO 27001 certified. No personally identifiable information was collected other than basic demographic descriptors, and all information, including IP addresses, was de-identified by the survey vendor prior to receipt of the responses by the authors. All data was password protected and stored on the survey vendor's database.

### 2.3 Statistical analysis

The survey vendor first analyzed data completeness and quality. All participants that started the survey completed the survey, and no participants were removed from the sample due to the survey vendor's data quality checks (e.g., poor open-ends, length of survey time less than 30% of median, and straight lining across multiple questions). 2,686 respondents entered the survey portal, but 103 did not or were unwilling to sign the research consent form. 2,232 respondents were removed, due to not meeting the inclusion criteria required to proceed with the survey.

Basic descriptive statistics were computed such as mean, median, and standard deviation for the response distributions. Furthermore, the frequency distributions were plotted as histograms to ensure the descriptive statistics served as appropriate representations of the data. Several statistical tests were applied to the data to determine statistical significance at *α* = 0.05 depending on the type of question (58). The Fischer Exact test was applied to contingency tables, especially when analyzing yes/no questions. The Mann–Whitney *U* test was applied to independent observations with ordinal, non-parametric data. Lastly, the chi-square test was used to analyze contingency tables resulting from *select all that apply* questions. Python version 3.12.7 (Python Software Foundation, Wilmington, Delaware, 2024) was used for statistical analysis.

## 3 Results

### 3.1 Description of the sample

The survey captured results from a diverse sample of respondents (Table 1), including 41% rural (*n* = 145) and 59% non-rural (*n* = 206) survey participants. Respondents could select multiple racial/ethnic categories. Sixty-five percent identified as white (not Hispanic/Latino), 14% Hispanic/Latino, 26% Black or African American, 2% Asian, and 3% American Indian/Alaska Native. The rural population was predominately white (80%). Age groups and gender were well represented, with no single age group (18–29 years, 30–39 years, 40–49 years, 50–59 years, 60 years or older) comprising more than 32% of the sample. Sixty percent of survey respondents identified as women, 40% identified as men, and 1% identified as transgender or non-binary. Sixty-nine percent were living with anxiety, 56% depression, 16% post-traumatic stress disorder, 13% bipolar disorder, 10% schizophrenia, 10% substance use disorder, 7% attention deficit disorder, 2% “Other,” 1% eating disorder, and 1% conduct disorder (respondents could select up to three options). Note that individuals with schizophrenia were oversampled to ensure adequate representation of serious mental illness.

**Table 1:** Demographic characteristics of survey participants (*n* = 351); *n* (%).

Demographics	Rural (*n* = 145)	Urban/Suburban (*n* = 206)	Total (*n* = 351)
Age			
Less than 18 years of age	0 (0%)	0 (0%)	0 (0%)
18–29 years of age	18 (12%)	34 (17%)	52 (15%)
30–39 years of age	41 (28%)	72 (35%)	113 (32%)
40–49 years of age	36 (25%)	48 (23%)	84 (24%)
50–59 years of age	37 (26%)	26 (13%)	63 (18%)
60 years of age or older	13 (9%)	26 (13%)	39 (11%)
Gender^a^			
Female	97 (67%)	112 (54%)	209 (60%)
Male	48 (33%)	92 (45%)	140 (40%)
Transgender or non-binary	0 (0%)	2 (1%)	2 (1%)
Prefer not to answer	0 (0%)	0 (0%)	0 (0%)
Race^a^			
Asian	1 (1%)	5 (2%)	6 (2%)
American Indian/Alaska Native	2 (1%)	9 (4%)	11 (3%)
Black or African American	20 (14%)	73 (35%)	93 (26%)
Hispanic/Latino	12 (8%)	38 (18%)	50 (14%)
Middle Eastern/North African	0 (0%)	0 (0%)	0 (0%)
Native Hawaiian/Other Pacific Islander	0 (0%)	1 (0%)	1 (0%)
White (*N*ot Hispanic/Latino)	116 (80%)	112 (54%)	228 (65%)
Other	0 (0%)	0 (0%)	0 (0%)
Ethnicity			
White (Not Hispanic/Latino)	113 (78%)	94 (46%)	207 (59%)
Non-White	32 (22%)	112 (54%)	144 (41%)
Mental Health Condition(s)^a^^,^^b^			
Depression	89 (61%)	108 (52%)	197 (56%)
Anxiety	106 (73%)	137 (67%)	243 (69%)
ADHD	9 (6%)	15 (7%)	24 (7%)
Bipolar Disorder	20 (14%)	27 (13%)	47 (13%)
Schizophrenia	10 (7%)	26 (13%)	36 (10%)
PTSD	26 (18%)	30 (15%)	56 (16%)
Conduct Disorder	1 (1%)	1 (0%)	2 (1%)
Substance Use Disorder	17 (12%)	19 (9%)	36 (10%)
Eating Disorder	2 (1%)	1 (0%)	3 (1%)
Other	4 (3%)	3 (1%)	7 (2%)
Rurality			
Rural	—	—	145 (41%)
Suburban	—	—	97 (28%)
Urban	—	—	109 (31%)

^a^ Total does not add to 100% due to participants being able to select more than one option.

^b^ Self-reported; respondents were asked “Have you been diagnosed with any of the following conditions (Please select all that apply)”.

### 3.2 Social determinants of health

In alignment with prior literature, rural respondents had lower incomes, lower education levels, and substantial Medicaid enrollment (Table 2) (13, 59–61). Rural respondents were significantly more likely to have lower household incomes than non-rural respondents (*p* = 0.004, Mann–Whitney *U* test). Specifically, the proportion of all respondents reporting an annual household income of <$35,000 (42%) was considerably higher than that reported nationally (21%), and more rural respondents fell in this income range (48% vs. 37% non-rural) (62). This difference may partly explain the higher proportion of rural respondents (60% vs. 46% non-rural) covered by Medicaid. Rural respondents also had lower levels of formal education compared to their non-rural counterparts (*p* = 0.042, Mann–Whitney *U* test), with 12% vs. 28% having a bachelor's degree or higher. A subset of the 144 participants who completed an optional free response question cited similar social determinants of health as contributors to limited care access.

**Table 2:** Social determinants of health characteristics of survey participants (*n* = 351); *n* (%).

Social determinants of health	Rural (*n* = 145)	Urban/Suburban (*n* = 206)	Total (*n* = 351)
Household income			
<$35,000	69 (48%)	77 (37%)	146 (42%)
$35,000–$75,000	55 (38%)	67 (33%)	122 (35%)
$75,000–$150,000	18 (12%)	43 (21%)	61 (17%)
>$150,000	3 (2%)	17 (8%)	20 (6%)
Do not know household income	0 (0%)	2 (1%)	2 (1%)
Education			
Less than high school	7 (5%)	10 (5%)	17 (5%)
High school/GED	53 (37%)	69 (33%)	122 (35%)
Some college, including technical school	67 (46%)	69 (33%)	136 (39%)
Bachelor's degree	15 (10%)	43 (21%)	58 (17%)
Advanced degree (Master's Doctorate, etc.)	3 (2%)	15 (7%)	18 (5%)
Insurance type^a^			
Insurance directly from an insurance company	19 (13%)	51 (25%)	70 (20%)
Insurance through a current or former employer or union	32 (22%)	52 (25%)	84 (24%)
Medicare	21 (14%)	39 (19%)	60 (17%)
Medicaid	87 (60%)	94 (46%)	181 (52%)
TRICARE or other military healthcare	1 (1%)	1 (0%)	2 (1%)
Veterans Affairs (VA)	4 (3%)	2 (1%)	6 (2%)
Indian Health Service	3 (2%)	1 (0%)	4 (1%)
I don’t have health insurance	1 (1%)	2 (1%)	3 (1%)
Don’t know/none of the above	2 (1%)	2 (1%)	4 (1%)

^a^ Total does not add to 100% due to participants being able to select more than one option.

“I’m unable to seek care because I’m on disability and have limited disposable income to pay for mental health care”—Respondent A

“I have 4 kids and I lost our home, it just makes it difficult to live and manage everything, then managing my mental health on top of that makes it harder”—Respondent B.

### 3.3 Barriers to care

To confirm whether distance to care was a unique challenge for rural communities, respondents were asked, “*How long is it by car to your closest in-person medical care?”* Rural respondents reported living significantly further from their nearest in-person medical care than non-rural respondents (*p* = 0.003, Mann–Whitney *U* test; Table 3). Ten percent of rural respondents vs. 0% of non-rural respondents lived more than 30 min from care, while more non-rural respondents lived only 5–10 min from care (40% vs. 28% rural).

**Table 3:** Survey participants’ responses to barriers to care (*n* = 351); *n* (%).

Survey responses	Rural (*n* = 145)	Urban/Suburban (*n* = 206)	Total (*n* = 351)
Barriers to care			
In the last year, have any of the following reasons made it difficult to receive care or made you stop seeking care (such as going to therapy or receiving prescribed medication) for your mental health condition?^a^			
I did not feel comfortable seeking care [for example, due to concerns about privacy or not being understood by my mental healthcare provider(s)]	14 (10%)	26 (13%)	40 (11%)
I was unable to find transportation and/or live too far from care	24 (17%)	28 (14%)	52 (15%)
It was too expensive and/or I did not have health insurance	18 (12%)	27 (13%)	45 (13%)
I did not have the time (for example, due to work, caregiving, or childcare)	20 (14%)	32 (16%)	52 (15%)
I could not find a mental healthcare provider and/or my mental healthcare provider was unavailable for an appointment	19 (13%)	22 (11%)	41 (12%)
I experienced technical difficulties (for example, I did not have reliable internet access for a telehealth visit)	14 (10%)	22 (11%)	36 (10%)
I had trouble managing my mental health condition(s) in between appointments	33 (23%)	40 (19%)	73 (21%)
I am always able to receive care	58 (40%)	87 (42%)	145 (41%)
Distance to care			
Less than 5 min	20 (14%)	35 (17%)	55 (16%)
5–10 min	41 (28%)	82 (40%)	123 (35%)
10–20 min	55 (38%)	68 (33%)	123 (35%)
20–30 min	15 (10%)	21 (10%)	36 (10%)
More than 30 min	14 (10%)	0 (0%)	14 (4%)

^a^ Total does not add to 100% due to participants being able to select more than one option.

In addition, a list of distinct mental healthcare access challenges was identified through a targeted literature review and consultation with the advising co-authors (Table 3). When asked, “*In the last year, have any of the following reasons made it difficult to receive care or made you stop seeking care (such as going to therapy or receiving prescribed medication) for your mental health condition?”* rural and non-rural participants reported individual barriers at a similar frequency (*p* = 0.754, Chi-square test). Notably, about 60% of all respondents faced at least one barrier to mental healthcare. The most common challenge across community types (21% of all respondents) was “I had trouble managing my mental health condition(s) in between appointments.” The least common (10% of all respondents) was, “I experienced technical difficulties.” However, this may have been due to selection bias since the survey was hosted online, and therefore required a baseline level of broadband access, technological capability, etc.

Respondents also reported challenges with distance to care/transportation (15%), time (15%), cost (13%), provider availability (12%), and comfort seeking care (11%). Participant free responses provided additional context about the cause and impact of such barriers, including missing appointments due to child-caring responsibilities or dependence on others for transportation, as well as feelings of isolation and a lack of support between appointments.

“Usually I have to depend on a friend to get to my appointments and sometimes they have work or important plans. I try to schedule for days when they are free, but another concern is having a doctor close to home who takes my insurance”—Respondent C.

“I’ve had to cancel previous appointments due to my child being sick and unable to find someone to watch her”—Respondent D.

### 3.4 Use of digital health

When asked, “*Do you use digital applications (also called ‘apps’ or software) or digital devices (like computers, smartphones, tablets, or smartwatches) to track or promote your physical or mental health?*” rural respondents were significantly less likely to have used any digital app or device for their physical or mental health (55% vs. 73%; *p* = 0.001, Fisher's exact test; Table 4). Because user engagement with digital therapies has been raised as a possible limitation, the survey also assessed the frequency of use (35). Twenty-eight percent of rural and 41% of non-rural respondents reported using the app/device at least once a day, while 21% of rural respondents and 29% of urban respondents reported using it at least once a week but less than once a day.

**Table 4:** Survey participants’ responses on digital health familiarity (*n* = 351); *n* (%).

Survey responses	Rural (*n* = 145)	Urban/Suburban (*n* = 206)	Total (*n* = 351)
Digital health use			
Do you use digital applications (also called “apps” or software) or digital devices (like computers, smartphones, tablets, or smartwatches) to track or promote your physical or mental health?			
Yes	80 (55%)	150 (73%)	230 (66%)
No	65 (45%)	56 (27%)	121 (34%)
How often do you use digital applications or digital devices to track or promote your physical or mental health?			
At least once a day	40 (28%)	85 (41%)	125 (36%)
At least once a week, but less than once a day	31 (21%)	59 (29%)	90 (26%)
At least once a month, but less than once a week	8 (6%)	3 (1%)	11 (3%)
Less than once a month	1 (1%)	3 (1%)	4 (1%)
Do not use	65 (45%)	56 (27%)	121 (34%)
Familiarity with Digital Mental Health Apps			
How familiar are you with digital apps for mental health treatment?^a^			
I have used a digital app for mental health treatment	48 (33%)	106 (51%)	154 (44%)
I have heard or seen an advertisement for digital apps for mental health treatment	60 (41%)	85 (41%)	145 (41%)
I know someone who has used a digital app for mental health treatment	35 (24%)	41 (20%)	76 (22%)
I am completely unfamiliar with digital apps for mental health treatment	31 (21%)	33 (16%)	64 (18%)
(*For participants that have used a digital app*) How did you first find out about the digital app?			
It was prescribed, ordered, or recommended to me by my mental healthcare provider (s)	15 (10%)	24 (12%)	39 (11%)
I saw an advertisement	7 (5%)	13 (6%)	20 (6%)
I saw it on social media	9 (6%)	17 (8%)	26 (7%)
Someone I know tried it and recommended it	2 (1%)	16 (8%)	18 (5%)
I found it while searching for treatment options online or on the app store	8 (6%)	23 (11%)	31 (9%)
My insurance offered it as a benefit	7 (5%)	10 (5%)	17 (5%)
My employer offered it as a benefit	0 (0%)	2 (1%)	2 (1%)
Other	0 (0%)	1 (0%)	1 (0%)
Did not use a digital mental health app	97 (67%)	100 (49%)	197 (56%)

^a^ Total does not add to 100% due to participants being able to select more than one option.

In addition to establishing baseline familiarity with digital health more broadly, this research sought to assess familiarity with applications used to treat mental health conditions. Rural respondents were more likely to be completely unfamiliar with digital apps for mental health treatment (21% vs. 16% non-rural) and were significantly less likely to already be using one (33% vs. 51%; *p* = 0.001, Fisher's exact test).

### 3.5 Respondent perceptions of PDTs

**Figure 1:**
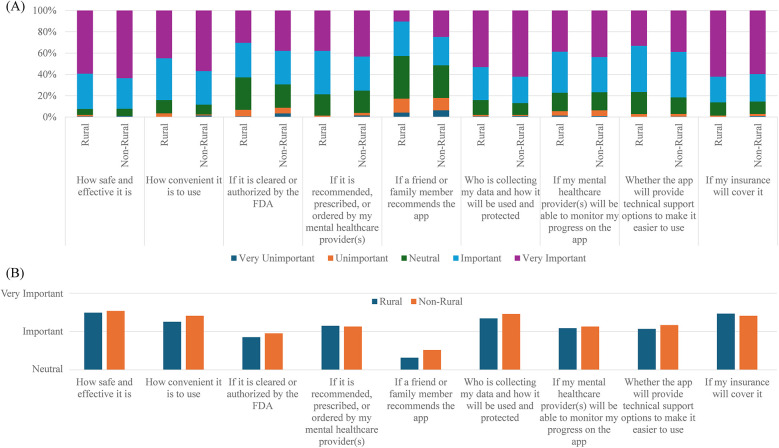
Responses to the question what would be important to know about the app wore using it? (1 = very unimportant, 2 = unimportant, 3 = neutral, 4 = important, 5 = very important). **(A)** Stacked bar chart of all responses separated by rural (*n* = 145) and non-rural (*n* = 206) survey participants. **(B)** The average response for rural and non-rural survey participants. Notably, “if a friend or family member recommends the app” was the least important feature. General trends were similar for the two groups across features.

This study aimed to assess potential users' perspectives on the features of PDTs. When provided with a detailed description of PDTs and illustrative examples, respondents were asked to rate features about the app that would be important to know prior to use. There were minimal differences in how rural and non-rural respondents ranked each variable, and most variables ranked similarly in importance. Convenience, safety and efficacy, insurance coverage, user data collection/protection, FDA clearance, and prescription by a provider were all considered “Important” (Figure 1). Notably, one factor ranked as less important was whether a friend or family member recommended the app.

This research also sought to gain an in-depth understanding of respondents' perceptions towards two defining PDT characteristics: FDA clearance and prescription by a mental healthcare provider. Both rural and urban/suburban respondents valued FDA clearance, with 68% of all respondents being more likely to trust the safety and efficacy of an FDA cleared app (Table 5). Provider endorsement was also highly valued, with 59% of respondents being very likely and 38% of respondents being somewhat likely to use a digital app that was recommended by their mental healthcare provider and available by prescription only. Rural and non-rural participants did not differ significantly in their responses (*p* = 0.480, Mann–Whitney *U* Test). Overall, about 97% of respondents were likely to use a PDT recommended by their provider.

**Table 5:** Survey participants’ perceptions of PDTs (*n* = 351); *n* (%).

Survey responses	Rural (*n* = 145)	Urban/Suburban (*n* = 206)	Total (*n* = 351)
PDT likelihood to use			
If a digital app is Food and Drug Administration (FDA) cleared or authorized, are you more likely to trust its safety and efficacy?			
Yes	96 (66%)	142 (69%)	238 (68%)
No	12 (8%)	14 (7%)	26 (7%)
Neutral	25 (17%)	43 (21%)	68 (19%)
I don’t know	12 (8%)	7 (3%)	19 (5%)
If a digital app was recommended by your mental healthcare provider(s) and available by prescription only, how likely are you to use it to help treat your mental health condition?			
Very likely	82 (57%)	125 (61%)	207 (59%)
Somewhat likely	59 (41%)	74 (36%)	133 (38%)
Somewhat unlikely	0 (0%)	6 (3%)	6 (2%)
Very unlikely	4 (3%)	1 (0%)	5 (1%)
Alleviation of barriers			
Which challenges do you think a safe and effective mental health focused digital app would be able to address the most?^a^			
Not feeling comfortable seeking care (for example, due to concerns about privacy or not being understood by my provider)	48 (33%)	63 (31%)	111 (32%)
Inability to find transportation and/or living too far from care	48 (33%)	61 (30%)	109 (31%)
Not having the time (for example, due to work, caregiving, or childcare)	34 (23%)	71 (34%)	105 (30%)
Not finding a mental healthcare provider and/or my provider is unavailable for an appointment	42 (29%)	67 (33%)	109 (31%)
Managing my mental health condition (s) in between appointments with my mental healthcare provider(s)	76 (52%)	109 (53%)	185 (52%)
It would not address any of these challenges	12 (8%)	26 (13%)	38 (11%)

^a^ Total does not add to 100% due to participants being able to select more than one option.

Finally, the survey assessed whether PDTs can help alleviate unmet needs. When asked what challenges a safe and effective mental health app could address, 89% of all respondents thought it could address at least one challenge from a list of five barriers probed earlier in the survey. The majority of respondents (52%) thought that the app could help manage their mental health condition between appointments, which was also the most reported barrier. About one third of respondents also thought PDTs could potentially address challenges related to comfort seeking care (32% of participants), transportation/distance from care (31%), provider availability (31%), and time (30%).

“I am doing a lot better now, but managing my mental health condition in between appointments was very challenging previously. Being at home alone was almost more than I could handle. I would have welcomed the app at that time. I think it is a great idea.”—Respondent E.

## 4 Discussion

In this study of 351 U.S. adults living with a mental health condition, clear barriers to adequate mental healthcare and high interest in PDTs were observed across community types. The results of this advocacy-led, person-centered survey demonstrate that PDTs are a promising new therapeutic option with the potential to overcome the diverse factors impeding treatment access, particularly those impacting rural communities. Though elements of this study's sample and methods—namely, digital access bias and rurality self-reporting—may limit generalizability to broader real-world contexts, the findings offer important insights with implications for future research and clinical practice.

While community-agnostic barriers like finding transportation, lacking time, and trouble affording care were widely experienced, rural respondents faced the added challenges of longer distances to care, lower incomes, less formal education, and high reliance on Medicaid in alignment with prior literature (13, 59–61). These widely recognized social determinants of mental healthcare access and quality represent a collective and intersectional burden for rural communities who might uniquely benefit from the time and resources saved with remote treatment options (6, 63).

Despite the need for remote therapies, rural respondents were significantly less likely to have used any digital app or device for their physical or mental health and were less likely to be familiar with digital apps for mental health treatment, echoing prior findings of lower mental health app utilization in rural vs. urban populations (35, 37). This divide in awareness and adoption may reflect limited access to traditional mental healthcare, since most respondents who used digital health apps were introduced to them by their providers (6). Consequently, facilitating access to prescribing physicians will be critical for rural people with mental health conditions to benefit as much as their non-rural counterparts, especially for prescription-only PDTs. Facilitation could entail efforts to scale up telehealth and other digital care interfaces alongside culturally relevant PDT awareness campaigns for providers and people living with a mental health condition (64, 65). Though broadband access and adoption in rural communities is still below that of non-rural communities, recent 5-year data indicates that broadband use has been increasing among rural people of all ages, presenting a critical opportunity for digital physician interactions and PDT use to grow in parallel (10).

This study was unique in that it specifically probed on potential users' perceptions of the differentiating features of PDTs: FDA clearance and prescription by a physician. Respondents had higher trust in an app with FDA clearance, viewing this as an indicator of safety and efficacy. “Trust” has been shown to be a key facilitator of digital app use, contextualizing the importance of this finding (36). FDA clearance was also considered “Important” alongside being recommended, prescribed, or ordered by a mental health provider, reinforcing the physician's role as a key intermediary, as detailed elsewhere (64, 65). Though clinician sentiment was not within the scope of this study, additional research should assess whether rural mental health providers' awareness, reservations, or levels of interest surrounding PDTs are aligned with those of rural people living with mental health conditions. Survey respondents also highly valued safety and efficacy, signaling the need for providers to communicate the robust clinical standards that distinguish PDTs from other digital health tools (25).

In contrast to prior research identifying negative sentiment towards mental health apps among rural communities (37), rural and non-rural respondents in our survey were equally excited about the prospect of PDTs. Frequent engagement among the 55% of respondents already using apps suggests they are well-received and helpful to those who have access. Further, rural and non-rural respondents' acknowledgement of PDTs' ability to overcome a variety of existing barriers implies that PDTs could address unmet needs in ways that are meaningful to users. Longitudinal and behavioral data are needed to confirm these interpretations, and future research might consider exploring whether interest in PDTs correlates with actual adoption, as well as whether PDT use improves long-term access to care, treatment satisfaction, and mental health outcomes.

This study has several limitations, primarily the online nature of the survey and the use of an elective surveying service, which potentially skewed recruitment towards individuals with at least some level of internet access, greater digital literacy, and a higher likelihood of using PDTs. This may also explain why technical difficulties were the least frequently reported barrier. Future research should leverage traditional surveying methods and a sample with low digital literacy to deeply explore whether factors like smartphone ownership, perceived ease of use, and broadband access alters receptivity among the rural population. Preference for a care-seeking population also likely skewed the sample towards those generally being more open to treatment whether through traditional or non-traditional means.

Although community type was defined with recognizable, plain-language definitions, self-reported rurality is another inherent limitation due to potential social desirability bias or misinterpretation by the respondent. This may have impacted the representativeness of the rural and non-rural groups, and future research could use alternative metrics for rurality or link individual survey data with zip codes to verify rurality. The connection between rurality and other social determinants of health may have also been a confounding factor for the observed rural/non-rural differences. Future studies and analyses could further explore the interplay between rurality and specific social determinants of health (*e.g.*, income and education) on access barriers and perspectives towards PDTs (13, 59–61).

The predominately white rural sample was not adequately representative of all races and ethnicities, which may limit generalizability and restrict capture of the intersectional challenges experienced by disparate groups within the rural community. The representativeness of the broader sample relative to the United States as a whole is another limitation, given potential geographic differences in payer coverage of mental healthcare, quality of care, and access to care.

To minimize respondent burden, the facilitators and barriers explored were by no means exhaustive, and additional value drivers not included in this study have been evaluated elsewhere (31, 35). Note, the study was not designed for preference testing of the most important PDT features, which will be an important area for future research. Specifically, due to the complexity of coverage and pricing, particularly with different insurance types, this research did not focus on out-of-pocket costs, which could be a key driver of real-world use (31). Additionally, this person-centered approach captured potential end-users' perspectives in isolation from the abundant policy and reimbursement hurdles that currently complicate PDT uptake (21). As such, high interest in PDTs among our sample does not guarantee real-world adoption, but it does support deeper consideration of the contextual factors shaping PDT uptake. These include coverage across various insurance types; education, treatment planning, and workflow management among providers; as well as the availability of culturally sensitive resources that enhance awareness of PDTs, particularly in rural areas (26, 31, 66, 67). More broadly, campaigns to expand the availability and affordability of internet access alongside digital literacy will be critical to facilitating equitable PDT uptake, particularly in rural areas (26, 31). In light of these variables, important complements to this and future research are published implementation science studies and related frameworks for integrating digital therapies into routine care (15, 68–74). Rural-specific applications of such approaches should be explored in detail for PDTs.

In conclusion, this first-of-its-kind study identified persistent gaps in mental healthcare and gauged interest in PDTs among potential users. Rural respondents faced worse social determinants of health and the added barrier of greater distances to care. Although familiarity with digital health was low, particularly among rural individuals, high interest in FDA-cleared, physician prescribed mental health apps across community types indicates strong receptivity to mental health PDTs. Explicit recognition of this new modality's capacity to overcome treatment barriers underscores the potential for PDTs to democratize care and transcend the infrastructural, environmental, and sociocultural challenges facing underserved rural communities.

## Data Availability

The datasets presented in this article are not readily available because of ethical and privacy concerns related to publicly sharing the full data. The authors will make deidentified data available upon reasonable request. Requests to access the datasets should be directed to Junko Saber, junko.saber@innopiphany.com.
