# Supplementary material for: Assessing rural populations’ barriers to mental healthcare and perceptions towards prescription digital therapeutics: a cross-sectional survey
Source: Front Digit Health. 2025 Sep 4;7:1655446. doi: 10.3389/fdgth.2025.1655446 (PMC12443765; doi:10.3389/fdgth.2025.1655446)
Supplement: Supplementary file 1 [file Datasheet1.pdf]

Mental Health Patient Screener

**1.**	What Race/Ethnicity do you identify as? (Select one or more)<br>• White (Not Hispanic/Latino)<br>• Hispanic/Latino<br>• Black or African American<br>• Asian<br>• American Indian/Alaska Native<br>• Native Hawaiian/Other Pacific Islander<br>• Middle East/North African<br>• Other: ____
**2.**	Which of the following best describes the community you live in?<br>• Rural – a small town or the countryside<br>• Suburban – a residential area near a city<br>• Urban – a medium sized or large city
**3.**	Which of the following age groups are you in?<br>• Less than 18 years of age<br>• 18 to 29 years of age<br>• 30 to 39 years of age<br>• 40 to 49 years of age<br>• 50 to 59 years of age<br>• 60 years of age or older
**4.**	How long is it by car to your closest in-person medical care?<br>• Less than 5 minutes<br>• 5 to 10 minutes<br>• 10 to 20 minutes<br>• 20 to 30 minutes<br>• More than 30 minutes
**5.**	Have you been diagnosed with any of the following conditions? (Please select all that apply)<br>• Depression (Including Post-Partum Depression)<br>• Anxiety<br>• Attention Deficit Hyperactivity Disorder<br>• Bipolar Disorder<br>• Schizophrenia, Schizoaffective, or Another Primary Psychosis Disorder (First Episode Psychosis)<br>• Post-Traumatic Stress Disorder<br>• Conduct Disorder<br>• Substance Use Disorder<br>• Eating Disorder<br>• Other: _____
**6.**	In the last year, have you tried to access mental healthcare (for example, going to therapy or getting a prescription medication)?<br>• I tried and received mental healthcare in the last year.<br>• I tried accessing mental healthcare in the last year but was unable to receive care.<br>• I have not tried to access care for my mental health condition in the past year.
**7.**	*[Displayed if 'I tried and received mental healthcare in the last year.' Selected in Question 6]*<br>In the last year, when you received mental healthcare, how often did you get the help or support you needed?<br>• Never<br>• Sometimes

	• Usually<br>• Always
**8.**	Are you currently treating your previously selected mental health condition(s) with prescribed medication and/or therapy?<br>• Yes<br>• No
**9.**	Do you use digital applications (also called "apps" or software) or digital devices (like computers, smartphones, tablets, or smartwatches) to track or promote your physical or mental health?<br>• Yes<br>• No
**10.**	*[Displayed if 'Yes' selected in Question 9]* How often do you use digital applications (also called "apps" or software) or digital devices (like computers, smartphones, tablets, or smartwatches) to track or promote your physical or mental health?<br>• At least once a day<br>• At least once a week, but less than once a day<br>• At least once a month, but less than once a week<br>• Less than once a month
