# Supplementary material for: Assessing rural populations’ barriers to mental healthcare and perceptions towards prescription digital therapeutics: a cross-sectional survey
Source: Front Digit Health. 2025 Sep 4;7:1655446. doi: 10.3389/fdgth.2025.1655446 (PMC12443765; doi:10.3389/fdgth.2025.1655446)
Supplement: Supplementary file 2 [file Datasheet2.pdf]

Survey:

**1.**	Do you currently have any of the following types of health insurance?<br>• Insurance directly from an insurance company<br>• Insurance through a current or former employer or union<br>• Medicare (for people 65 and older or people with certain disabilities)<br>• Medicaid or any kind of government-assistance plan for those with low incomes or disability<br>• TRICARE or other military healthcare<br>• Veterans Affairs (VA)<br>• Indian Health Service<br>• I don't have health insurance<br>• Don't know / none of the above
**2.**	In general, how would you rate your overall mental or emotional health?<br>• Excellent<br>• Very Good<br>• Good<br>• Fair<br>• Poor
**3.**	In the past year, how did you communicate with your mental healthcare provider(s) such as your doctor, psychiatrist, or therapist? *Please select all that apply from the responses below.*<br>• Provider's Website or Patient Portal Messaging<br>• Phone Call<br>• Text Message<br>• Email<br>• In-Person<br>• Telehealth Video Call<br>• I did not communicate with a mental healthcare provider
**4.**	In the last year, have any of the following reasons made it difficult to receive care or made you stop seeking care (such as going to therapy or receiving prescribed medication) for your mental health condition? *Please select all that apply from the reasons below.*<br>• I did not feel comfortable seeking care (for example, due to concerns about privacy or not being understood by my mental healthcare provider(s))<br>• I was unable to find transportation and/or live too far from care<br>• It was too expensive and/or I did not have health insurance<br>• I did not have the time (for example, due to work, caregiving, or childcare)<br>• I could not find a mental healthcare provider and/or my mental healthcare provider was unavailable for an appointment<br>• I experienced technical difficulties (for example, I did not have reliable internet access for a Telehealth visit)<br>• I had trouble managing my mental health condition(s) in between appointments<br>• I am always able to receive care
**5.**	(*Displayed if more than one option was selected in Q4 and 'I am always able to receive care was not selected'*) Of the options you selected in Question 4, please rank them from 1 to X *(X being the number of selections from Q4)* with 1 being the largest challenge and X being the smallest challenge.<br>• I did not feel comfortable seeking care (for example, due to concerns about privacy or not being understood by my mental healthcare provider(s))<br>• I was unable to find transportation and/or live too far from care

	• It was too expensive and/or I did not have health insurance<br>• I did not have the time (for example, due to work, caregiving, or childcare)<br>• I could not find a mental healthcare provider and/or my mental healthcare provider was unavailable for an appointment<br>• I experienced technical difficulties (for example, I did not have reliable internet access)<br>• I had trouble managing my mental health condition(s) in between appointments
**6.**	How familiar are you with digital apps for mental health treatment? *Please select all that apply from the responses below.*<br>• I have used a digital app for mental health treatment<br>• I have heard or seen an advertisement for digital apps for mental health treatment<br>• I know someone who has used a digital app for mental health treatment<br>• I am completely unfamiliar with digital apps for mental health treatment
**7.**	(*Displayed if 'I have used a digital app for mental health treatment' was selected in Q6*) How did you first find out about the digital app? *Please indicate your answer below.*<br>• It was prescribed, ordered, or recommended to me by my mental healthcare provider(s)<br>• I saw an advertisement<br>• I saw it on social media<br>• Someone I know tried it and recommended it<br>• I found it while searching for treatment options online or on the app store<br>• My insurance offered it as a benefit<br>• My employer offered it as a benefit<br>• Other:______
For the remaining questions, assume that there is a new digital app available that you can have on any smartphone. The app has interactive activities and reminders that have been shown to help treat your mental health condition. For example:<br>• **Example 1:** You may complete short brain exercises (such as remembering emotions on faces) on the app 3 times a week for 6 weeks to feel less depressed.<br>• **Example 2:** An app may teach you coping skills for substance use disorder through lessons that you read or watch, plus daily check-ins to measure your progress.	
**8.**	What would be important to know about the app before using it? *Please slide.*<br>*(Note: The Food and Drug Administration (FDA) is a US government agency which regulates the safety of food, drugs, medical devices, and other products by reviewing them for safety and effectiveness)* (1 = Very Unimportant, 2 = Unimportant, 3 = Neutral, 4 = Important, 5 = Very Important)<br>• How safe and effective it is.<br>• How convenient it is to use<br>• If it is cleared or authorized by the Food and Drug Administration (FDA)<br>• If it is recommended, prescribed, or ordered by my mental healthcare provider(s)<br>• If a friend or family member recommends the app<br>• Who is collecting my data and how it will be used and protected<br>• If my mental healthcare provider(s) will be able to monitor my progress on the app<br>• Whether the app will provide technical support options to make it easier to use<br>• If my insurance will cover it
**9.**	If a digital app is **Food and Drug Administration (FDA) cleared or authorized**, are you more likely to trust its safety and efficacy?<br>• Yes<br>• No

	• Neutral<br>• I don't know
**10.**	If a digital app was **recommended by your mental healthcare provider(s) and available by prescription only**, how likely are you to use it to help treat your mental health condition? *Please indicate your response below.*<br>• Very Likely<br>• Somewhat Likely<br>• Somewhat Unlikely<br>• Very Unlikely
**11.**	Which challenges do you think a safe and effective mental health focused digital app would be able to address the <u>**most**</u>? *Please select up to 3 challenges below.*<br>• Not feeling comfortable seeking care (for example, due to concerns about privacy or not being understood by my provider)<br>• Inability to find transportation and/or living too far from care<br>• Not having the time (for example, due to work, caregiving, or childcare)<br>• Not finding a mental healthcare provider and/or my provider is unavailable for an appointment<br>• Managing my mental health condition(s) in between appointments with my mental healthcare provider(s)<br>• It would not address any of these challenges
**12.**	How excited are you about the possibility of a digital app that is cleared or authorized by the Food and Drug Administration (FDA) and prescribed or ordered by your mental health provider to help treat your mental health condition?<br>• Very Excited<br>• Somewhat Excited<br>• Not Excited
**13.**	***OPTIONAL*: You previously selected (*populated with the previously selected Rank 1 response from Q5*) as the biggest challenge to your mental healthcare. Would you like to add anything about how this challenge has affected you or are there any other challenges you would like to mention?
**14.**	How would you describe your gender identity? *Please select all that apply*.<br>• Man<br>• Woman<br>• Non-binary<br>• Transgender<br>• Other<br>• Prefer not to answer
**15.**	What is the combined income of all your household members?<br>• <$35,000<br>• $35,000 - $75,000<br>• $75,000 - $150,000<br>• >$150,000<br>• Do not know household income
**16.**	What is the highest level of school that you've completed?<br>• Less than High School<br>• High School/GED<br>• Some college, including technical school<br>• Bachelor's Degree<br>• Advanced Degree (Master's, Doctorate, etc.)
